# Epigallocatechin-3-Gallate and PEDF 335 Peptide, 67LR Activators, Attenuate Vasogenic Edema, and Astroglial Degeneration Following Status Epilepticus

**DOI:** 10.3390/antiox9090854

**Published:** 2020-09-11

**Authors:** Ji-Eun Kim, Hana Park, Min-Jeong Jeong, Tae-Cheon Kang

**Affiliations:** 1 Department of Anatomy and Neurobiology, College of Medicine, Hallym University, Chuncheon 24252, Korea; jieunkim@hallym.ac.kr (J.-E.K.); M19050@hallym.ac.kr (H.P.); 20183646@hallym.ac.kr (M.-J.J.); 2 Institute of Epilepsy Research, College of Medicine, Hallym University, Chuncheon 24252, Korea

**Keywords:** AKT, AQP4, blood–brain barrier (BBB), endothelial cell, endothelial nitric oxide synthase (eNOS), extracellular signal-regulated kinase 1/2 (ERK1/2), NADPH oxidase, NU335, p38 mitogen-activated protein kinase (p38 MAPK)

## Abstract

Non-integrin 67-kDa laminin receptor (67LR) is involved in cell adherence to the basement membrane, and it regulates the interactions between laminin and other receptors. The dysfunction of 67LR leads to serum extravasation via blood-brain barrier (BBB) disruption. Polyphenol (–)-epigallocatechin-3-*O*-gallate (EGCG) and pigment epithelium-derived factor (PEDF) bind to 67LR and inhibit neovascularization. Therefore, in the present study, we investigated the effects of EGCG and NU335, a PEDF-derive peptide, on BBB integrity and their possible underlying mechanisms against vasogenic edema formation induced by status epilepticus (SE, a prolonged seizure activity). Following SE, both EGCG and NU335 attenuated serum extravasation and astroglial degeneration in the rat piriform cortex (PC). Both EGCG and NU335 reversely regulated phosphatidylinositol 3 kinase (PI3K)/AKT–eNOS (endothelial nitric oxide synthase) mediated BBB permeability and aquaporin 4 (AQP4) expression in endothelial cells and astrocytes through the p38 mitogen-activated protein kinase (p38 MAPK) and extracellular signal-regulated kinase 1/2 (ERK1/2) signaling pathways, respectively. Furthermore, EGCG and NU335 decreased p47Phox (a nicotinamide adenine dinucleotide phosphate oxidase subunit) expression in astrocytes under physiological and post-SE conditions. Therefore, we suggest that EGCG and PEDF derivatives may activate 67LR and its downstream effectors, and they may be considerable anti-vasogenic edema agents.

## 1. Introduction

Status epilepticus (SE), defined as a prolonged seizure activity, is a common neurological emergency with considerable morbidity and mortality. In addition, SE is one of the high risk factors of developing acquired epilepsy [1]. As well as neuronal death, SE results in serum extravasation into the brain parenchyma (vasogenic edema) due to the blood-brain barrier (BBB) breakdown [2,3]. Vasogenic edema is widely considered to be detrimental for outcome following SE and leads to the paroxysmal neuronal discharge, neuroinflammation, and astroglial dysfunction, which is involved in epileptogenesis [4,5]. In addition, vasogenic edema results in undesirable secondary complications in various neurological diseases including an abrupt increase in intracranial pressure, abnormal neuronal excitability, and gray and white matter injuries [6,7,8,9]. SE-induced BBB breakdown is relevant to the generation of nitric oxide (NO) by endothelial nitric oxide synthase (eNOS) in endothelial cells and reactive oxygen species (ROS) by nicotinamide adenine dinucleotide phosphate (NADPH) oxidase in astrocytes, respectively [10]. Thus, it is likely that SE may result in BBB dysfunctions via endothelial-astroglial interactions through the nitrosactive and oxidative stresses. However, the underlying mechanisms and the related signaling pathways for eNOS and NADPH oxidase activations during SE-induced vasogenic edema formation are still unknown.

Endothelial cells reciprocally connect and constitute tight junctions through extracellular adhesion proteins (basement membrane) and form BBB with astrocytes and pericytes. Laminin is a major glycoprotein component of the basement membrane in vessels, which regulates neovascularization, vascular dilation, and its integrity [11,12]. The laminin functions are mediated by the integrins and the non-integrin 67-kDa laminin receptor (67LR). Since 67LR arises from a 32–33 kDa precursor, this receptor is also called 32 kDa laminin binding protein (LBP), 32 kDa laminin binding protein precursor (LBP-32, 37LRP), p40, and ribosomal protein SA (RPSA) [13,14,15,16]. 67LR plays a role in cell adherence to basement membrane and stabilizes or modulates the binding of laminin to other receptors [17,18]. Furthermore, 67LR modulates various signaling pathways in cellular functions [19]. Indeed, 67LR regulates p38 mitogen-activated protein kinase (p38 MAPK), extracellular signal-regulated kinase 1/2 (ERK1/2), and c-Jun N-terminal kinase (JNK) [20]. Recently, we have reported that SE decreases 67LR expression concomitant with BBB breakdown in the rat piriform cortex (PC). Similar to SE, 67LR neutralization evokes vasogenic edema by reducing aquaporin 4 (AQP4) expression and increasing p38 MAPK/vascular endothelial growth factor (VEGF) activities in the PC [21,22,23]. Therefore, it is likely that 67LR may play an important role in the maintenance of BBB integrity. 

On the other hand, polyphenol (–)-epigallocatechin-3-*O*-gallate (EGCG) binds to 67LR and induces apoptosis in cancer cells [24,25,26]. Similar to EGCG, the full-length pigment epithelium-derived factor (PEDF) and its derived peptides bind to 67LR and inhibit neovascularization [27,28]. Interestingly, EGCG protects endothelial cells from apoptosis [29,30], and it reduces vascular permeability in vitro [31,32]. Furthermore, PEDF attenuates BBB disruption induced by ischemic stroke [33,34,35]. With respect to these previous reports, it is likely that both EGCG and PEDF may regulate 67LR-mediated BBB integrity, although their possible underlying mechanisms remain incompletely understood. 

Here, we demonstrate for the first time that EGCG and PEDF peptide (NU335) attenuated SE-induced vasogenic edema formation by regulating phosphatidylinositol 3 kinase (PI3K)/AKT-mediated BBB permeability and AQP4 expression in endothelial cells and astrocytes through p38 MAPK and ERK1/2 pathways, respectively. Furthermore, these anti-vasogenic edema effects were closely relevant to the inhibitions of eNOS and NADPH oxidase expression in endothelial cells and astrocytes, respectively. Therefore, our findings suggest that 67LR may play a protective role against oxidative and nitrosactive stresses following SE, and its downstream effectors may be considerable therapeutic targets, which have important implications for the development and use of EGCG and PEDF derivatives as anti-vasogenic edema agents.

## 2. Materials and Methods 

### 2.1. Experimental Animals and Chemicals

Adult male Sprague–Dawley (SD) rats (7 weeks old) were housed on standard light–dark cycle (12-h light/dark cycle), temperature (22 ± 2 °C), and humidity (55 ± 5%) with rat chow pellets and tap water continuously available. All experimental protocols were approved by the Institutional Animal Care and Use Committee of Hallym University (Chuncheon, Korea, Hallym 2017-54, 19 Febrary 2017 and Hallym 2018-2, 26 April 2018). All reagents were obtained from Sigma-Aldrich (St. Louis, MO, USA), except as noted.

### 2.2. Surgery 

Under isoflurane anesthesia (3% induction, 1.5–2% for surgery, and 1.5% maintenance in a 65:35 mixture of N_2_O:O_2_), rats were implanted a brain infusion kit 1 (Alzet, Cupertino, CA, USA) into the right lateral ventricle (1 mm posterior; 1.5 mm lateral; −3.5 mm depth to the bregma) and connected with an Alzet 1003D or 1007D osmotic pump (Alzet, Cupertino, CA, USA) containing (1) vehicle, (2) EEGC (50 μM), and (3) NU335 (1 μM, kindly gifted from Professor Jack Henkin, Northwestern University, USA). Three days after surgery, Alzet 1003D-infused animals were used for Western blot and immunohistochemistry. Alzet 1007D-infused rats were also implanted with a monopolar stainless steel electrode (Plastics One, Roanoke, VA, USA) into the left dorsal hippocampus using the following coordinates: −3.8 mm posterior; 2.0 mm lateral; −2.6 mm depth. An electrode was secured to the exposed skull with dental acrylic. Three days after surgery, Alzet 1007D-infused animals was induced with SE by lithium chloride (LiCl)-pilocarpine.

### 2.3. SE Induction and EEG Recording

Rats were injected intraperitoneally with LiCl (127 mg/kg) 24 h prior to the administration of pilocarpine (30 mg/kg). Twenty minutes before pilocarpine injection, animals were given atropine methylbromide (5 mg/kg) to block the peripheral effect of pilocarpine. SE induction was stopped 2 h after pilocarpine injection by the administration of diazepam (10 mg/kg, i.p.). Diazepam was repeatedly administered as needed. To validate the effect of EGCG or NU335 on seizure susceptibility induced by pilocarpine, electroencephalogram (EEG) signals of electrode-implanted animals were measured with a DAM 80 differential amplifier (0.1–3000 Hz bandpass; World Precision Instruments, Sarasota, FL, USA). EEG activity was measured (2 h recording session), digitized (400 Hz), and analyzed using LabChart Pro v7 (AD Instruments, New South Wales, Australia). The time of seizure onset was defined as the time point showing paroxysmal discharges (4–10 Hz with 2 times higher amplitude than the basal level) that lasted more than 3 seconds. Spectrograms were also automatically calibrated using a Hanning sliding window with 50% overlap [21,22,23].

### 2.4. Tissue Processing

Three days after SE induction, animals were perfused transcardially with phosphate-buffered saline (PBS, pH 7.4) followed by 4% paraformaldehyde in 0.1 M phosphate buffer (PB, pH 7.4) under urethane anesthesia (1.5 g/kg intraperitoneally (i.p.)). The brains were removed and stored in the same fixative for 4 h. Subsequently, brains were shifted to PB) containing 30% sucrose at 4 °C for 2 days. Coronal sections (30 μm) were prepared with a cryostat, and consecutive sections were collected in six-well plates containing PBS. For Western blots, animals were rats were sacrificed by decapitation under urethane anesthesia. The PC was rapidly dissected out and homogenized in lysis buffer containing protease inhibitor cocktail (Roche Applied Sciences, Branford, CT, USA) and phosphatase inhibitor cocktail (PhosSTOP^®^, Roche Applied Science, Branford, CT, USA). The protein concentration in the supernatant was determined using a Micro BCA Protein Assay Kit (Pierce Chemical, Dallas, TX, USA).

### 2.5. Western Blot

Western blot was performed by the standard protocol. Briefly, sample proteins (10 μg) were separated on a Bis-Tris sodium dodecyl sulfate-poly-acrylamide electrophoresis gel (SDS-PAGE). Then, separated proteins were transferred to polyvinylidene fluoride membranes that were blocked overnight at 4 °C with 2% bovine serum albumin (BSA) in Tris-buffered saline (TBS; in mM 10 Tris, 150 NaCl, pH 7.5, and 0.05% Tween 20) and then incubated in primary antibodies (Table 1). Thereafter, the membranes were incubated for 1 h at room temperature in a solution containing horseradish peroxidase (HRP)-conjugated secondary antibodies. Immunoblots were quantified by membrane scanning in an ImageQuant LAS4000 system (GE Healthcare Korea, Seoul, Korea). Optical densities of proteins were calculated by the protein/β-actin ratio. The ratio of phosphoprotein to total protein was described as the phosphorylation level.

### 2.6. Immunohistochemistry

Free-floating sections were washed 3 times in PBS (0.1 M, pH 7.3). After blocking the endogenous peroxidase with 3% H_2_O_2_ and 10% methanol in PBS (0.1 M) for 20 min at room temperature, sections were incubated in 10% normal goat serum (Vector, Burlingame, CA, USA). Later, sections were incubated in biotinylated rat immunoglobulin G (IgG) and avidin-biotin complex (ABC) kit (Vector, #PK-6100, USA, diluted 1:200) and visualized by 3,3′-diaminobenzidine in 0.1 M Tris buffer. Some sections were incubated in a mixture of primary antibodies shown in Table 1 (in PBS containing 0.3% Triton X-100) at room temperature, overnight. For negative control, tissues were incubated in pre-immune serum instead of primary antibody. After washing 3 times, tissues were incubated with a fluorescein isothiocyanate (FITC)- or Cy3-conjugated secondary antibodies (Vector, Burlingame, CA, USA) for 1 h at room temperature.

### 2.7. Measurements of Serum Extravasation, GFAP-Deleted Area, and Fluorescent Intensity

The areas showing vasogenic edema and glial fibrillary acidic protein (GFAP, an astroglial marker) deletion (10 sections per each animal) were measured using AxioVision Rel. 4.8 software (Carl Zeiss Korea, Seoul, Korea) [23,36,37,38]. To quantify fluorescent intensity, sections (10 sections per each animal) were captured using an AxioImage M2 microscope. Thereafter, the mean intensity of phospho (p)-p38 MAPK, pERK1/2, pAKT, eNOS, p47Phox (a nicotinamide adenine dinucleotide phosphate oxidase subunit), and SMI-71 (an endothelial BBB marker) signals were measured using AxioVision Rel. 4.8 software. Fluorescent intensity measurements (10 sections per each animal) were represented as the number of a 256-gray scale and corrected by subtracting the average values of background noise obtained from 5 image inputs.

### 2.8. Statistical Analysis

The values on normality of results were evaluated using Shapiro–Wilk *W*-test. Data were analyzed by one-way ANOVA or repeated measure ANOVA to determine statistical significance. Newman–Keuls test was used for post hoc comparisons. A *p*  <  0.05 was considered to be statistically different.

## 3. Results

### 3.1. Effects of EGCG and NU335 Peptide on Seizure Susceptibility in Response to Pilocarpine

**Figure 1:**
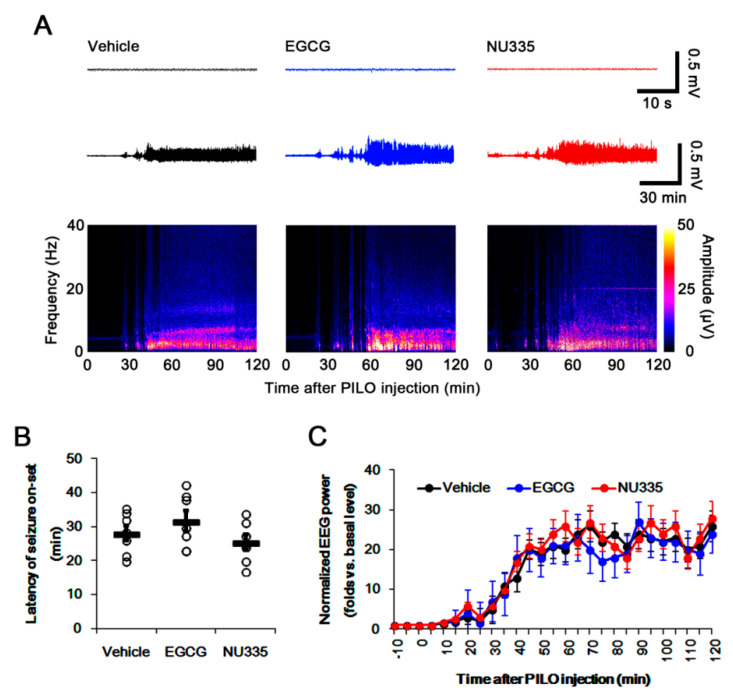
Effects of epigallocatechin-3-*O*-gallate (EGCG) and NU335 on seizure activity in response to pilocarpine. Neither EGCG nor NU335 affects seizure activity and seizure threshold in response to pilocarpine. (**A**) Representative baseline electroencephalogram (EEG) (upper traces), seizure activity (lower traces), and frequency-power spectral temporal maps in response to pilocarpine. (**B**) Quantification of latency of seizure onset in response to pilocarpine (*n* = 7, respectively). Open circles indicate each individual value. Horizontal bars indicate mean value. Error bars indicate S.E.M. (**C**) Quantification of total EEG power (seizure intensity) in response to pilocarpine (*n* = 7, respectively). Error bars indicate S.E.M.

First, we explored whether EGCG or NU335 influences susceptibility to SE induction. Both EGCG and NU335 did not result in behavioral seizures. As compared to vehicle, both EGCG and NU335 did not affect baseline EEG, latency of seizure onset, and seizure intensity in response to pilocarpine (Figure 1A–C). Therefore, these findings indicate that EGCG and NU335 may not influence the seizure susceptibility in response to pilocarpine. 

### 3.2. Effects of EGCG and NU335 Peptide on Serum Extravasation Following SE

**Figure 2:**
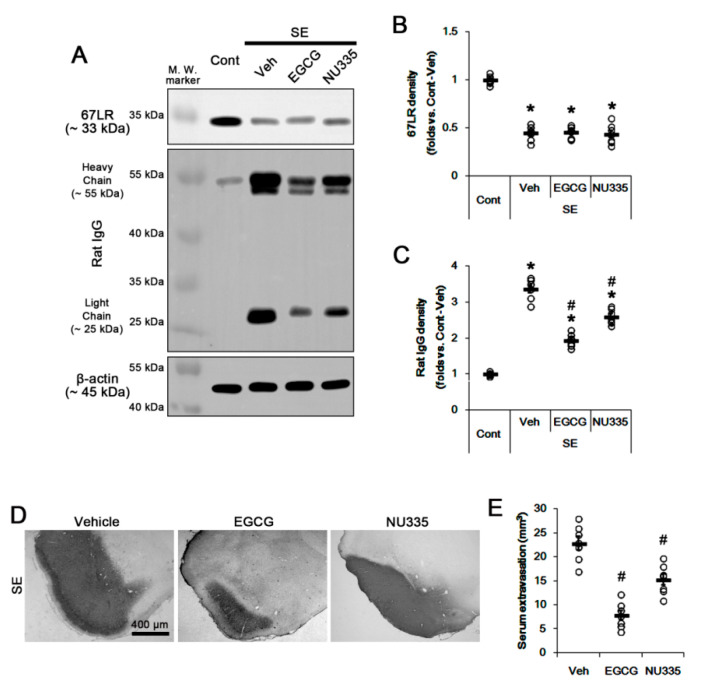
Effects of EGCG and NU335 on vasogenic edema formation in the piriform cortex (PC) following status epilepticus (SE). EGCG and NU335 do not affect SE-induced reductions in 67-kDa laminin receptor (67LR) expression. However, both EGCG and NU335 attenuate serum extravasation and vasogenic edema formation induced by SE. (**A**) Representative Western blot images for 67LR and rat immunoglobulin G( IgG) in the PC. (**B**,**C**) Quantitative values of the effect of EGCG and NU335 on 67LR (**B**) and serum extravasation (**C**) in the PC following SE (*n* = 7, respectively). Open circles indicate each individual value. Horizontal bars indicate mean value. Error bars indicate S.E.M. Significant differences are *^,#^ *p* < 0.05 vs. control (non-SE) animals and vehicle-treated SE-induced animals, respectively. (**D**) Representative photographs for vasogenic edema in the PC. (**E**) Quantitative values of the effects of EGCG and NU335 on serum extravasation. Open circles indicate each individual value. Horizontal bars indicate mean value. Error bars indicate S.E.M. Significant differences are * *p* < 0.05 vs. vehicle-treated animals (Student *t*-test).

Next, we investigated whether EGCG or NU335 affects 67LR expression and BBB permeability under post-SE conditions. Since vasogenic edema peaks in the PC at 3 days after SE [36], we explore the effects of EGCG and NU335 on vasogenic edema formation using 3-day post-SE animals. Following SE, 67LR expression was significantly reduced in the PC (*p* < 0.05 vs. control animals, one-way ANOVA, *n* = 7; Figure 2A,B). This SE-induced 67LR reduction was unaffected by EGCG or NU335 (Figure 2A,B). SE led to severe serum extravasation in the PC (*p* < 0.05 vs. control animals, one-way ANOVA, *n* = 7; Figure 2A,C). As compared to vehicle, EGCG and NU335 decreased SE-induced serum extravasation to 0.58- and 0.77-fold of vehicle level, respectively (*p* < 0.05 vs. vehicle, one-way ANOVA, *n* = 7; Figure 2A,C). EGCG and NU335 showed approximately 66% and 33% reduction in the volume of vasogenic edema in the PC, respectively (*p* < 0.05 vs. vehicle, one-way ANOVA, *n* = 7; Figure 2D,E). These findings indicate that EGCG and NU335 may ameliorate SE-induced vasogenic edema formation without altering 67LR expression and seizure susceptibility in response to pilocarpine.

### 3.3. Effects of EGCG and NU335 Peptide on Astroglial Damage under Physiological and Post-SE Conditions 

**Figure 3:**
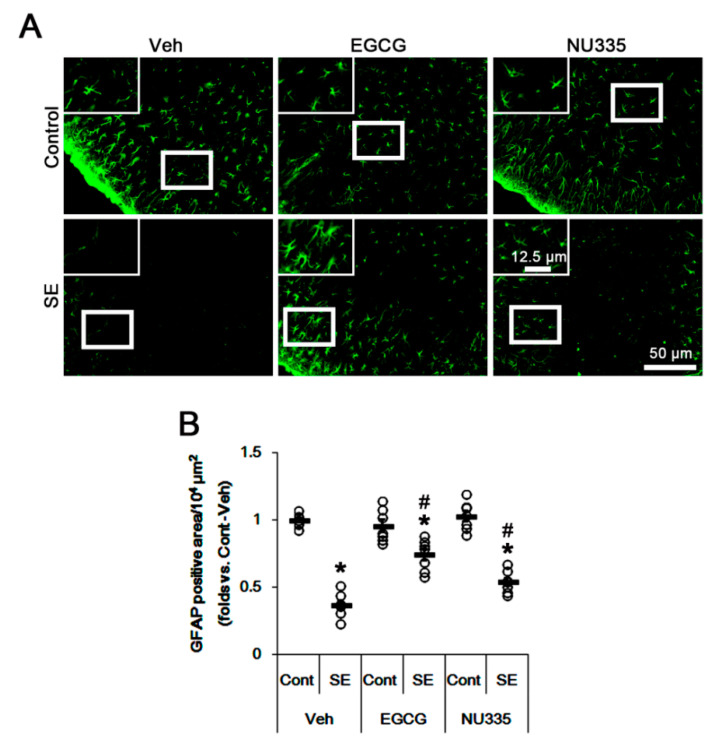
Astroglial responses to EGCG and NU335 in the PC under physiological and post-SE conditions. EGCG and NU335 do not lead to reactive astrogliosis or astroglial loss under physiological conditions. However, both EGCG and NU335 attenuate astroglial degeneration induced by SE. (**A**) Representative photos for glial fibrillary acidic protein (GFAP) in the PC under physiological (upper panels) and post-SE (lower panels) conditions. (**B**) Quantitative values of the effect of EGCG and NU335 on the GFAP-deleted area in the PC following SE (*n* = 7, respectively). Open circles indicate each individual value. Horizontal bars indicate mean value. Error bars indicate S.E.M. Significant differences are *^,#^ *p* < 0.05 vs. control (non-SE) animals and vehicle-treated animals, respectively.

Since astroglial loss is closely relevant to vasogenic edema formation [21,36,38], we explored the effects of EGCG and NU335 on astroglial viability under physiological and post-SE conditions. As compared to vehicle, EGCG and NU335 did not lead to reactive astrogliosis and astroglial loss in the PC under physiological conditions. Thus, there was no difference in the GFAP-positive area in the PC among the three groups (Figure 3A,B). Following SE, the GFAP-positive area was reduced to approximately 0.37-fold of control level in the PC due to massive astroglial loss (*p* < 0.05, one-way ANOVA, *n* = 7, respectively; Figure 3A,B). EGCG and NU335 attenuated the GFAP-deleted area in the PC; thus, the GFAP-positive area was 0.74- and 0.54-fold of the control level (*p* < 0.05, one-way ANOVA, *n* = 7, respectively; Figure 3A,B). These findings indicate that EGCG and NU335 may reduce astroglial vulnerability to SE in the PC. 

### 3.4. The Effects of EGCG and NU335 on Phosphorylations of p38 MAPK and ERK1/2 

**Figure 4:**
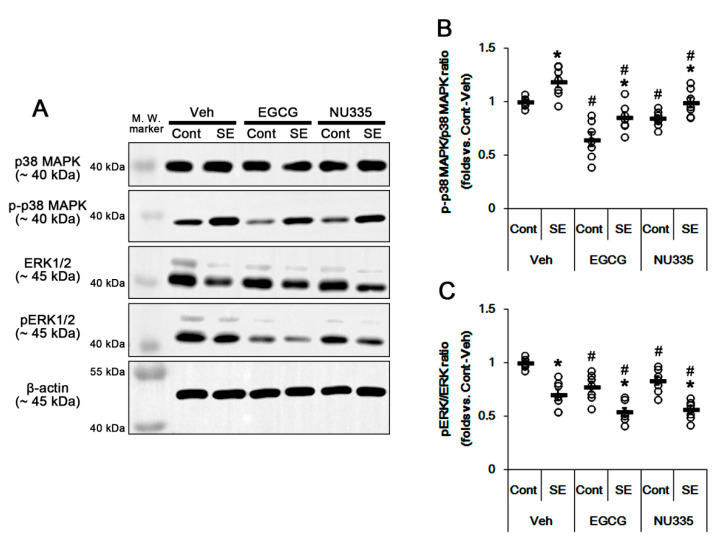
Effects of EGCG and NU335 on p38 mitogen-activated protein kinase (p38 MAPK) and extracellular signal-regulated kinase (ERK) activities in the PC under physiological and post-SE conditions. SE increases p38 MAPK, but not ERK1/2, activity in the PC. EGCG and NU335 decrease p38 MAPK and ERK activities under physiological and post-SE conditions. (**A**) Representative Western blot images for p38 MAPK, p-p38 MAPK, ERK1/2, and pERK1/2 in the PC. (**B**,**C**) Quantitative values of the effect of EGCG and NU335 on p38 MAPK (**B**) and ERK1/2 phosphorylation levels in the PC (*n* = 7, respectively). Open circles indicate each individual value. Horizontal bars indicate the mean value. Error bars indicate S.E.M. Significant differences are *^,#^ *p* < 0.05 vs. control (non-SE) animals and vehicle-treated animals, respectively.

The blockade of 67LR functionality increases p38 MAPK and ERK1/2 activities [20,21,22,23]. Thus, we explored the effects of EGCG and NU335 on their phosphorylations (activities). Under physiological condition, neither EGCG nor NU335 affected p38 MAPK and ERK1/2 expressions in the PC. However, EGCG reduced p38 MAPK and ERK1/2 phosphorylations to approximately 0.65- and 0.77-fold of the vehicle level, respectively (*p* < 0.05 vs. vehicle, one-way ANOVA; *n* = 7, respectively; Figure 4A,C). In addition, NU335 decreased them to approximately 0.85- and 0.83-fold of the vehicle level (*p* < 0.05 vs. vehicle, one-way ANOVA; *n* = 7, respectively; Figure 4A,C). 

**Figure 5:**
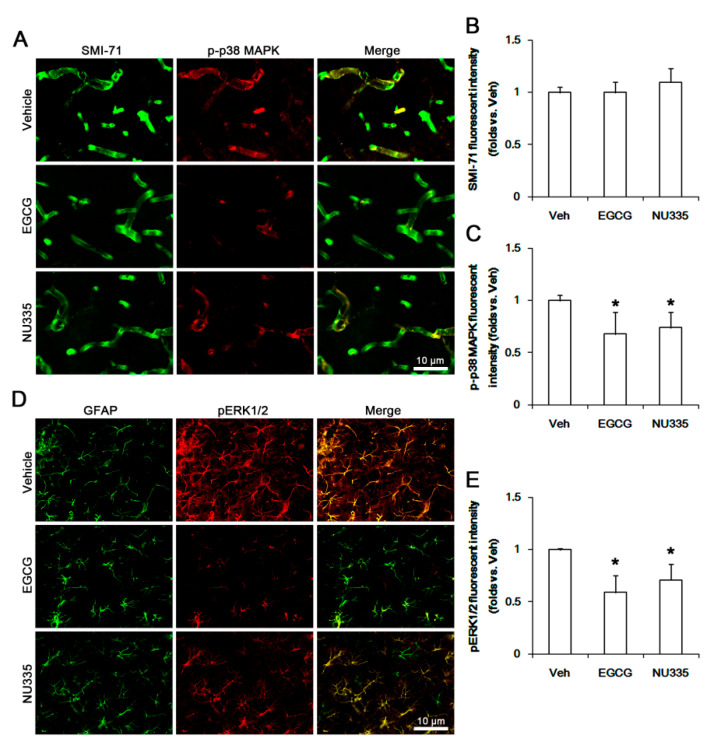
Effects of EGCG and NU335 on endothelial p-p38 MAPK and astroglial pERK level in the PC under physiological condition. EGCG and NU335 abolish p38 MAPK and pERK levels in endothelial cells and astrocytes, respectively. (**A**) Representative images for endothelial p-p38MAPK signals in the PC. (**B**,**C**) Quantification of the effect of EGCG and NU335 on SMI-71 (an endothelial blood–brain barrier (BBB) antigen) and p-p38MAPK fluorescent intensities (*n* = 7, respectively). Error bars indicate S.E.M. Significant differences are * *p* < 0.05 vs. vehicle-treated animals. (**D**) Representative images for astroglial pERK signals in the PC. (**E**) Quantification of the effect of EGCG and NU335 on pERK1/2 fluorescent intensity (*n* = 7, respectively). Error bars indicate S.E.M. Significant differences are * *p* < 0.05 vs. vehicle-treated animals.

Immunohistochemical studies revealed that both EGCG and NU335 did not affect the SMI-71 (an endothelial BBB marker) expression level in the PC (*p* < 0.05 vs. vehicle, one-way ANOVA; *n* = 7, respectively; Figure 5A,B). However, EGCG reduced p-p38 MAPK and pERK1/2 signals to 0.68- and 0.59-fold of the vehicle level in the PC. In addition, NU335 diminished them to 0.74- and 0.71-fold of the vehicle level, respectively (*p* < 0.05 vs. vehicle, one-way ANOVA; *n* = 7, respectively; Figure 5A,C–E). These alterations in p-p38 MAPK and pERK1/2 signals were preferentially observed in endothelial cells and astrocytes, respectively (Figure 5A).

Following SE, the p38 MAPK phosphorylation ratio was increased to 1.2-fold of the control level, but its expression was not increased in the PC (*p* < 0.05 vs. control animals, one-way ANOVA; *n* = 7, respectively; Figure 4A,B). In contrast, SE reduced ERK1/2 expression and its phosphorylation level. The ERK1/2 phosphorylation ratio was 0.7-fold the control level (*p* < 0.05 vs. control animals, one-way ANOVA; n = 7, respectively; Figure 4A,C). EGCG attenuated SE-induced p38 MAPK phosphorylation to approximately 0.86-fold the control level (*p* < 0.05 vs. vehicle, one-way ANOVA; *n* = 7, respectively; Figure 4A,B). EGCG reinforced the reduction in ERK1/2 phosphorylation ratio to 0.54-fold the control level following SE (*p* < 0.05 vs. vehicle, one-way ANOVA; *n* = 7, respectively; Figure 4A,C). Similar to EGCG, NU335 ameliorated the SE-induced p38 MAPK phosphorylation ratio 0.99-fold of the control level (*p* < 0.05 vs. vehicle, one-way ANOVA; *n* = 7, respectively; Figure 4A,B). NU335 also further reduced the ERK1/2 phosphorylation ratio to 0.56-fold the control level following SE (*p* < 0.05 vs. vehicle, one-way ANOVA; *n* = 7, respectively; Figure 4A,C). Therefore, these findings indicate that EGCG and NU335 may inhibit p38 MAPK and ERK1/2 phosphorylations in the PC under physiological and post-SE conditions. 

### 3.5. The Effects of EGCG and NU335 on PI3K/AKT Phosphorylation and AQP4 Expression 

**Figure 6:**
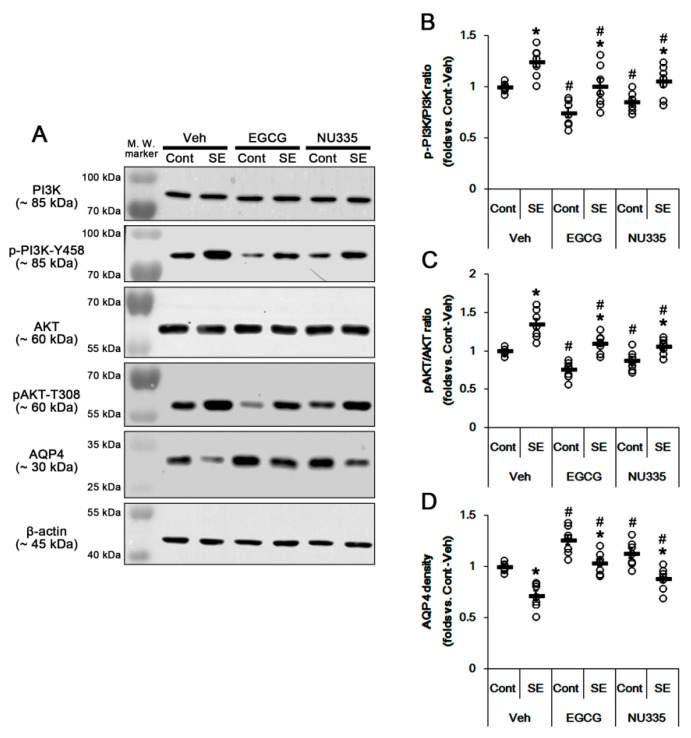
Effects of EGCG and NU335 on phosphatidylinositol 3 kinase (PI3K)/AKT activities and aquaporin 4 (AQP4) expression level in the PC under physiological and post-SE conditions. SE increases PI3K/AKT activities, but it decreases the AQP4 protein level in the PC. EGCG and NU335 decrease PI3K/AKT activities under physiological and post-SE conditions. (**A**) Representative Western blot images for PI3K, p-PI3K, AKT, AKT, and AQP4 in the PC. (**B**–**D**) Quantitative values of the effect of EGCG and NU335 on p-p38 MAPK (**B**), pAKT and AQP4 levels in the PC (*n* = 7, respectively). Open circles indicate each individual value. Horizontal bars indicate mean value. Error bars indicate S.E.M. Significant differences are *^,#^ *p* < 0.05 vs. control (non-SE) animals and vehicle-treated animals, respectively.

p38 MAPK-mediated PI3K/AKT activations are involved in SE-induced serum extravasation [39,40]. In addition, PI3K and ERK1/2 reciprocally regulate each other [23,41,42,43]. Indeed, ERK1/2-mediated AKT phosphorylation downregulates AQP4 expression in astrocytes [44]. Thus, we explored if EGCG and NU335 affect PI3K/AKT activities and AQP4 expression in the PC. Under physiological conditions, EGCG did not influence PI3K and AKT expression levels in the PC (Figure 6A–C). However, EGCG reduced pPI3K-Y458 and pAKT-T308 phosphorylation levels to 0.74- and 0.76-fold the vehicle level, respectively (*p* < 0.05 vs. vehicle, one-way ANOVA; *n* = 7, respectively; Figure 6A–C). NU335 also decreased PI3K and AKT phosphorylation levels to 0.85- and 0.88-fold the vehicle level, respectively (*p* < 0.05 vs. vehicle, one-way ANOVA; *n* = 7, respectively; Figure 6A–C). Furthermore, EGCG and NU335 increased AQP4 expression to 1.26- and 1.13-fold the vehicle level (*p* < 0.05 vs. vehicle, one-way ANOVA; *n* = 7, respectively; Figure 6A,D). 

**Figure 7:**
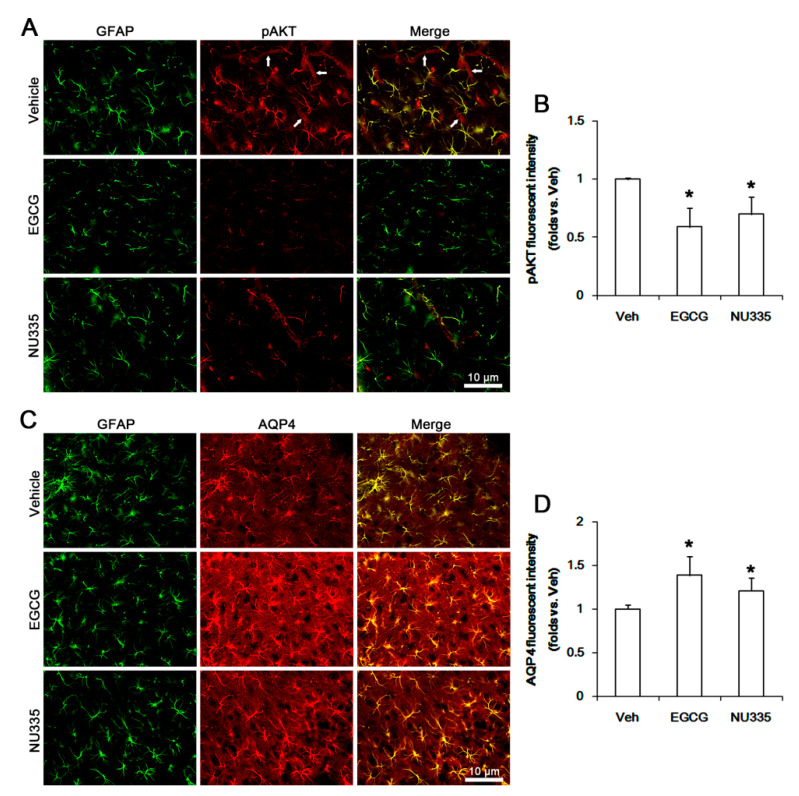
Effects of EGCG and NU335 on pAKT and AQP4 levels in the PC under physiological condition. pAKT signals are observed in endothelial cells (arrows, upper panels in A) and astrocytes. EGCG and NU335 decrease pAKT levels in endothelial cells and astrocytes. However, they increase astroglial AQP4 expression. (**A**) Representative images for pAKT signals in the PC. (**B**) Quantification of the effect of EGCG and NU335 on pAKT fluorescent intensities (*n* = 7, respectively). Error bars indicate S.E.M. Significant differences are * *p* < 0.05 vs. vehicle-treated animals. (**C**) Representative images for AQP4 signals in the PC. (**D**) Quantification of the effect of EGCG and NU335 on AQP4 fluorescent intensity (*n* = 7, respectively). Error bars indicate S.E.M. Significant differences are * *p* < 0.05 vs. vehicle-treated animals.

Immunofluorescent study demonstrated that pAKT signals were observed in endothelial cells and astrocytes (Figure 7A). EGCG and NU335 reduced pAKT fluorescent intensity in astrocytes and endothelial cells (*p* < 0.05 vs. vehicle, one-way ANOVA; *n* = 7, respectively; Figure 7A,B), while they enhanced AQP4 expression level in astrocytes (*p* < 0.05 vs. vehicle, one-way ANOVA; *n* = 7, respectively; Figure 7C,D). Following SE, PI3K and AKT were increased to 1.25- and 1.35-fold the control level (*p* < 0.05 vs. control animals, one-way ANOVA; *n* = 7, respectively; Figure 6A–C). However, AQP4 expression was reduced to 0.72-fold the control level in the PC (*p* < 0.05 vs. control animals, one-way ANOVA; *n* = 7, respectively; Figure 6A,D). EGCG and NU335 abrogated the SE-induced increases in PI3K and AKT phosphorylations to vehicle-treated control animal level (*p* < 0.05 vs. vehicle, one-way ANOVA; *n* = 7, respectively; Figure 6A–C). They also attenuated the SE-induced decrease in AQP4 expression (*p* < 0.05 vs. vehicle, one-way ANOVA; *n* = 7, respectively; Figure 6A,D). These findings indicate that EGCG and NU335 may ameliorate vasogenic edema by inhibiting the alterations in PI3K/AKT-mediated BBB permeability and AQP4 expression induced by SE.

### 3.6. The Effects of EGCG and NU335 on eNOS and NADPH Oxidase Expression 

**Figure 8:**
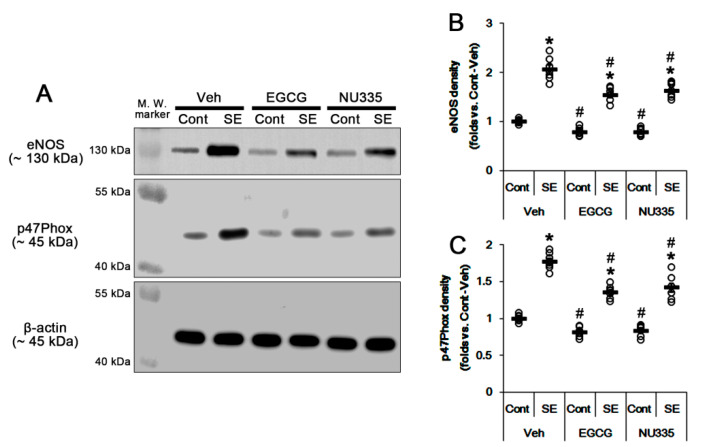
Effects of EGCG and NU335 on endothelial nitric oxide synthase (eNOS) and p47Phox (a nicotinamide adenine dinucleotide phosphate oxidase subunit) expression levels in the PC under physiological and post-SE conditions. SE increases eNOS and p47Phox expression levels in the PC. EGCG and NU335 reduce eNOS and p47Phox expression levels under physiological and post-SE conditions. (**A**) Representative Western blot images for eNOS and p47Phox in the PC. (**B**,**C**) Quantitative values of the effect of EGCG and NU335 on eNOS (**B**) and p47Phox in the PC (*n* = 7, respectively). Open circles indicate each individual value. Horizontal bars indicate mean value. Error bars indicate S.E.M. Significant differences are *^,#^ *p* < 0.05 vs. control (non-SE) animals and vehicle-treated animals, respectively.

SE leads to nitrosactive and oxidative stresses in endothelial cell and astrocytes, respectively [10]. SE triggers the production of NO derived from endothelial nitric oxide synthase (eNOS) in endothelial cells, which increases BBB permeability [45,46]. SE also activates astroglial NADPH oxidase, which is the major source of ROS. p47phox plays an important role in the assembly of the NADPH complex that is composed of p40, p47, p67, gp91, and P22phox [47,48]. Indeed, apocynin, an NADPH oxidase inhibitor, attenuates SE-induced vasogenic edema [10]. Thus, we investigated whether EGCG and NU355 affect eNOS and p47Phox expression. Under physiological conditions, EGCG decreased eNOS and p47Phox expression levels to 0.78- and 0.81-fold the vehicle level, respectively (*p* < 0.05 vs. vehicle, one-way ANOVA; *n* = 7, respectively; Figure 8A–C). NU335 also reduced eNOS and p47Phox expression levels to 0.77- and 0.83-fold the vehicle level, respectively (*p* < 0.05 vs. vehicle, one-way ANOVA; *n* = 7, respectively; Figure 8A–C).

**Figure 9:**
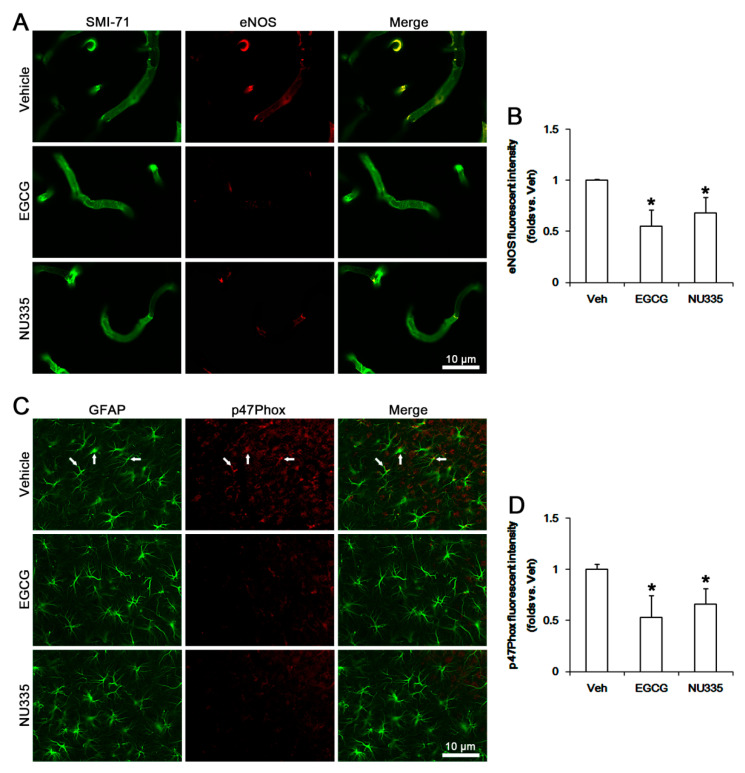
Effects of EGCG and NU335 on eNOS and p47Phox levels in the PC under physiological condition. eNOS signals are detected in endothelial cells. P47Phox expressions are observed in neurons and astrocytes (arrows, upper panels in C). EGCG and NU335 abolish eNOS and p47Phox expressions in endothelial cells and astrocytes, respectively. (**A**) Representative images for eNOS signals in the PC. (**B**) Quantification of the effect of EGCG and NU335 on eNOS fluorescent intensity (*n* = 7, respectively). Error bars indicate S.E.M. Significant differences are * *p* < 0.05 vs. vehicle-treated animals. (**C**) Representative images for astroglial p47Phox signals in the PC. (**D**) Quantification of the effect of EGCG and NU335 on p47Phox fluorescent intensity (*n* = 7, respectively). Error bars indicate S.E.M. Significant differences are * *p* < 0.05 vs. vehicle-treated animals.

Immunofluorescent study demonstrated that EGCG and NU335 reduced eNOS expression in endothelial cells (*p* < 0.05 vs. vehicle, one-way ANOVA; *n* = 7, respectively; Figure 9A,B). In addition, EGCG and NU335 declined p47Phox expression in astrocytes (*p* < 0.05 vs. vehicle, one-way ANOVA; *n* = 7, respectively; Figure 9C,D). Consistent with our previous study [10], eNOS and p47Phox expression were increased to 2.06- and 1.77-fold the control level following SE (*p* < 0.05 vs. control animals, one-way ANOVA; *n* = 7, respectively; Figure 8A–C). EGCG abrogated the SE-induced increases in eNOS and p47Phox levels to 1.54- and 1.35-fold the vehicle-treated control animal level (*p* < 0.05 vs. vehicle, one-way ANOVA; *n* = 7, respectively; Figure 8A–C). NU335 also ameliorated SE-induced increases in eNOS and p47Phox levels to 1.63- and 1.42-fold the vehicle-treated control animal level (*p* < 0.05 vs. vehicle, one-way ANOVA; *n* = 7, respectively; Figure 8A–C). These findings indicate that EGCG and NU335 may mitigate SE-induced vasogenic edema by abolishing endothelial NO synthesis and astroglial ROS generation. 

## 4. Discussion

**Figure 10:**
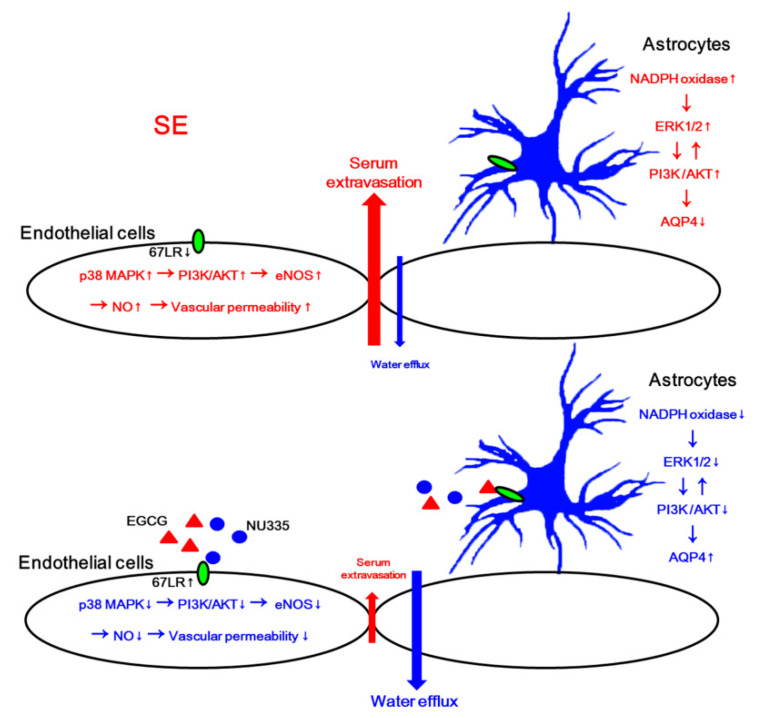
Scheme of the underlying mechanisms of EGCG and NU335 against vasogenic edema formation. The reduced 67LR functionality induced by SE activates p38 MAPK–PI3K/AKT–eNOS and nicotinamide adenine dinucleotide phosphate (NADPH) oxidase–ERK1/2–PI3K/AKT signaling pathways in endothelial cells and astrocytes, respectively. Subsequently, the increased nitric oxide (NO) generation enhances BBB permeability. In addition, astroglial AKT activation reduces AQP4 expression, leading to the impaired water efflux from the brain parenchyma, which worsens vasogenic edema. EGCG and NU335 attenuate vasogenic edema formation via 67LR activation that plays inhibitory roles in these p38 MAPK and NADPH oxidase-mediated signaling pathways in endothelial cells and astrocytes.

The major findings in the present study are that EGCG and NU335 attenuated SE-induced vasogenic edema formation. In addition, EGCG and NU335 reversely regulated PI3K/AKT-mediated BBB permeability and AQP4 expression in endothelial cells and astrocytes, which were modulated by p38 MAPK–ERK1/2 and NADPH oxidase–ERK1/2 signaling pathways, respectively (Figure 10). 

The BBB plays an important role in the maintenance of the microenvironment for brain functions by separating the brain from the systemic circulatory systems and regulating the transports of intravascular substances into the brain [49]. Under pathophysiological conditions, BBB is disrupted, and the subsequent serum-derived molecules are leaked in the brain parenchyma. This serum extravasation leads to neuronal excitability and neuroinflammation [50,51,52,53]. Thus, the preservation of BBB integrity is one of the therapeutic strategies to inhibit undesirable consequences from brain insults. 

67LR is a laminin receptor that regulates cell adhesion and migration [19,20]. 67LR modulates SE-induced vasogenic edema through the p38 MAPK–PI3K/AKT–eNOS signaling pathway [39,40]. Indeed, the blockade of 67-kDa LR increases vascular permeability by p38 MAPK-mediated VEGF activation [21]. Furthermore, SB202190 (a p38 MAPK inhibitor) alleviates serum extravasation induced by SE and 67LR neutralization [21,22]. Recently, we have reported that the blockade of 67LR functionality decreases BBB integrity due to p38 MAPK-mediated AKT activation in endothelial cells [21,22,23]. However, we could not directly provide whether 67LR integrates this gliovascular interaction. In the present study, EGCG attenuated SE-induced vasogenic edema in the PC without altering the seizure susceptibility to pilocarpine. This effect of EGCG on serum extravasation was relevant to the inhibition of p38 MAPK-mediated PI3K/AKT activity and eNOS upregulation in endothelial cells under physiological and post-SE conditions. Indeed, EGCG modulates the PI3K/AKT–eNOS signaling pathway [30], which increases BBB permeability in the PC [21,22,23,40]. Since EGCG activates 67LR-mediated signaling pathways [54,55], our findings indicate that EGCG-mediated 67LR activation may attenuate vasogenic edema formation induced by SE. 

PEDF is a 50-kDa protein secreted by the retinal pigment epithelium in the eye as well as other tissues [56]. PEDF has anti-angiogenic, anti-tumorigenic, anti-inflammatory, anti-oxidative, neurotrophic, neuroprotective, and anti-vasopermeability properties [57,58,59,60,61]. In the present study, NU335, a novel bioactive PEDF-derived peptide [28], ameliorated SE-induced vasogenic edema in the PC. Similar to EGCG, NU335 inhibited the p38 MAPK–PI3K/AKT–eNOS signaling pathway in endothelial cells under physiological and post-SE conditions. Therefore, these findings indicate that NU335 may also attenuate p38 MAPK/PI3K/AKT–eNOS-mediated vasogenic edema formation following SE. However, the effects of PEDF on p38 MAPK activation and vascular permeability are still controversial. PEDF increases VEGF expression and p38 MAPK phosphorylation [62,63], which lead to endothelial barrier dysfunction in human umbilical vein endothelial cells (HUVECs) in vitro [64]. It has been also reported that PEDF decreases AKT phosphorylation without altering p38 MAPK activity in cancer cells [65]. In contrast, PEDF inhibits p38 MAPK in the retina [66]. Furthermore, *PEDF^−/^^−^* mice exhibit increase in vessel permeability [67]. These discrepancies may be due to receptor-specific responses to PEDF: PEDF and its derivatives bind to membrane proteins including adipose triglyceride lipase (ATGL), VEGF receptor 1, patatin-like phospholipase domain containing 2, and 67LR [27,68,69,70,71]. In contrast to 67LR, ATGL activation increases p38 MAPK phosphorylation, which is required for PEDF-induced tumor-necrosis factor (TNF) production [62]. Considering the NU335 interactions with 67LR and p38 MAPK-mediated serum extravasation induced by 67LR neutralization, our findings suggest that NU335 may inhibit SE-induced vasogenic edema formation by enhancing 67LR-mediated p38 MAPK inhibition. 

In the present study, we found that both EGCG and NU335 decreased ERK1/2 phosphorylation under physiological conditions. These findings are consistent with previous studies demonstrating that soluble laminin and EGCG decrease ERK1/2 phosphorylation [20,72,73,74,75]. Furthermore, PEDF diminished ERK1/2 phosphorylation [65,66]. The present data showed that the reduced ERK1/2 phosphorylation was restricted to astrocytes. EGCG and NU335 also diminished AKT phosphorylation under physiological conditions. As a result of the interaction between the PI3K and ERK1/2 pathways [23,41,42,43], it is not surprising that EGCG- and NU335-induced ERK1/2 inhibition may decrease AKT activity in astrocytes. Indeed, U0126 (an ERK1/2 inhibitor) and wortmannin (a PI3K inhibitor) decreased AKT phosphorylation in astrocytes following 67LR neutralization [23]. Furthermore, both EGCG and NU335 increased the AQP4 expression level in astrocytes. Since AKT activation downregulates AQP4 expression [23,44], it is likely that AKT may be a common downstream effector for PI3K and ERK1/2 to inhibit AQP4 expression in astrocytes. However, the roles of ERK1/2 in the regulation of AQP4 expression have been still controversial: ERK1/2 activation inhibits [76], upregulates [77], or cannot influence AQP4 expression [78]. In our previous study [21,22,23], we found that 67LR neutralization diminishes AQP4 expression, which is abrogated by wortmannin, U0126, and 3CAI (an AKT inhibitor). Thus, our findings suggest that EGCG- or NU335-induced ERK1/2 inhibition may increase AQP4 expression through the AKT-mediated indirect pathway rather than the direct pathway. Thus, it is likely that the presence of AKT-mediated regulation may result in the discrepancies of AQP4 regulation by ERK1/2. 

In previous studies, we have reported that astroglial degeneration/dysfunction is not a primary cause of vasogenic edema formation [21,22,23]. In addition, AQP4 deletion cannot evoke serum extravasation [6,79]. However, AQP4 deletion or its inhibition in astrocytes deteriorates vasogenic edema due to the impaired vasogenic water elimination from parenchyma to vessels, which subsequently worsens astroglial degeneration [6,36,79]. Recently, we have also reported that U0126 aggravated SE-induced vasogenic edema and astroglial loss in the PC, while 3CAI alleviated these post-SE events [23]. On the contrary, the present study demonstrates that EGCG and NU335 attenuated the SE-induced AQP4 reduction in the PC, which is accompanied by decreasing ERK1/2 and AKT phosphorylations. In addition, EGCG and NU335 ameliorated SE-induced astroglial loss in the PC. These findings suggest that the EGCG- and NU335-induced inhibition of the ERK1/2-AKT signaling pathway may increase astroglial viability, which would be involved in the prevention of SE-induced AQP4 downregulation. On the other hand, astroglial NADPH oxidase activation reduces AQP4 expression and astroglial viability [10]. Interestingly, ROS generation from NADPH oxidase triggers downstream pathways for the ERK1/2 and AKT, but not p38 MAPK phosphorylations in astrocytes [80,81]. NADPH oxidase-mediated ROS activate ERK1/2 and AKT, but not p38 MAPK [82]. In the present study, ECGC and NU335 reduced astroglial p47Phox expression under physiological and post-SE conditions. Thus, it is likely that 67LR-mediated NADPH oxidase inhibition may decrease ERK1/2 and AKT phosphorylations, which would upregulate AQP4 expression. Indeed, EGCG inhibits the activities of NADPH oxidase [83] and ERK1/2 via 67LR [75,84]. PEDF also abrogates ERK1/2 and NADPH oxidase activities [85,86,87]. Thus, our findings suggest that EGCG and NU335 may lead to astroglial AQP4 upregulation by 67LR-medaited NADPH oxidase inhibition, which would decrease ERK1/2-AKT activities. 

As aforementioned, most SE models show serum extravasation in the brain parenchyma, which is involved in ictogenesis and epileptogenesis [2,3,4,5]. Pilocarpine-induced SE generally leads to more intense damage in more areas of the brain than kainic acid (KA)-induced SE of similar duration [88]. In addition, KA results in cytotoxic edema in the hippocampus and piriform cortex where pilocarpine induces severe serum extravasation, although it develops vasogenic edema in the frontal cortex, thalamus, and the striatum [10,22,36,89,90]. Based on these features, we believe that a pilocarpine model may be better to evaluate the efficacy of EGCG and NU335 against SE-induced serum extravasation than the KA model. Using the same rationale, our findings suggest that EGCG and NU335 may be effective and possibly strong agents against vasogenic edema.

On the other hand, various insults such as stroke and head trauma also evoke vasogenic edema that increases intracranial pressure, leading to fatal conditions [91]. Furthermore, retinal vascular permeability is increased in diabetic retinopathy and exudative macular degeneration that cause blindness [28,92,93]. Currently, corticosteroids and agents blocking VEGF are the predominant treatment options for brain edema and retina macular edema, respectively. Unfortunately, both corticosteroids and anti-VEGF therapy induce adverse effects [94]. Thus, it is likely that EGCG and/or NU335 may be attractive and novel therapeutic agents for vasogenic cerebral edema and retinal macular edema. However, EGCG reaches the brain parenchyma even at a very low concentration [95]. In addition, the permeability of NU335 into the BBB is unknown. Therefore, the development of novel BBB-permeable derivatives of EGCG and NU335 would be needed for clinical trials concerning prevention treatment for vasogenic edema.

## 5. Conclusions

To the best of our knowledge, the present data reveal for the first time that EGCG and NU335, as 67LR activators, attenuated SE-induced vasogenic edema formation by inhibiting p38 MAPK–PI3K/AKT–eNOS axis in endothelial cells. In addition, they increased AQP4 expression by abrogating the NADPH oxidase–ERK1/2–PI3K/AKT–AQP4 signaling pathway in astrocytes (Figure 10). Therefore, our findings suggest that the 67LR activation may be one of the therapeutic strategies for the prevention of vasogenic edema formation and its complications induced by nitrosactive and oxidative stresses.

## Figures and Tables

**Table 1:** Primary antibodies and lectin used in the present study.

Antigen	Host	Manufacturer (Catalog Number)	Dilution Used
67LR	Rabbit	Abcam (#ab133645)	1:1000 (WB)
AKT	Rabbit	Cell signaling (#9272)	1:1000 (WB)
AQP4	Rabbit	Alomone labs (#AQP-004)	1:5000 (WB) 1:200 (IH)
eNOS	Rabbit	Abcam (ab66127)	1:1000 (WB) 1:500 (IH)
ERK1/2	Rabbit	Biorbyt (Orb160960)	1:2000 (WB)
GFAP	Mouse	Millipore (#MAB3402)	1:5000 (IH)
p-p38 MAPK	Rabbit	Abbiotec (#251256)	1:200 (WB) 1:50 (IH)
p38 MAPK	Rabbit	Cell signaling (#9212)	1:1000 (WB)
p47Phox	Rabbit	Abbiotec (252159)	1:1000 (WB) 1:200 (IH)
pAKT-T308	Rabbit	Cell signalling (#9275)	1:1000 (WB) 1:50 (IH)
pERK1/2	Rabbit	Bioss (bs-3330R)	1:1000 (WB) 1:100 (IH)
PI3K	Rabbit	Cell signaling (#4292)	1:1000 (WB)
pPI3K-Y458	Rabbit	Cell signaling (#4228)	1:1000 (WB)
Rat IgG	Goat	Vector (#BA-9400)	1:2000 (WB) 1:200 (IH)
SMI-71	Mouse	Covance (#SMI-71R)	1:1000 (IH)
β-actin	Mouse	Sigma (#A5316)	1:5000 (WB)

IH: Immunohistochemistry; WB: Western blot.
